# miRNAs in cerebrospinal fluid associated with Alzheimer's disease: a systematic review and pathway analysis using a data mining and machine learning approach

**DOI:** 10.1002/alz70856_104445

**Published:** 2025-12-26

**Authors:** Jessica Diniz Pereira, Lívia Cristina Ribeiro Teixeira, Izabela Mamede Costa Andrade da Conceição, Michelle Teodoro Alves, Marcelo Rizzatti Luizon, Adriano Alonso Veloso, Paulo Caramelli, Karina Braga Gomes

**Affiliations:** ^1^ Faculty of Pharmacy ‐ Federal University of Minas Gerais, Belo Horizonte, Minas Gerais, Brazil; ^2^ Faculty of Medicine ‐ Federal University of Minas Gerais, Belo Horizonte, Minas Gerais, Brazil; ^3^ Institute of Biological Sciences ‐ Federal University of Minas Gerais, Belo Horizonte, Minas Gerais, Brazil; ^4^ HEMOMINAS, Belo Horizonte, Minas Gerais, Brazil; ^5^ Institute of Exact Sciences ‐ Federal University of Minas Gerais, Belo Horizonte, Minas Gerais, Brazil; ^6^ Federal University of Minas Gerais, Belo Horizonte, Minas Gerais, Brazil

## Abstract

**Background:**

Alzheimer's disease (AD) is the most common type of dementia and accounts for around 70% of reported cases. MicroRNAs are small non‐coding RNAs with highly conserved sequences, involved in gene modulation and several molecular mechanisms. miRNAs can be found in cerebrospinal fluid (CSF) and serve as potential biomarkers for AD diagnosis. This study aimed to perform an *in silico* analysis of miRNA expression in the CSF of AD patients compared to cognitively healthy controls and to conduct an analysis of the biological pathways regulated by these miRNAs.

**Method:**

Employing a machine learning approach, we identified the most differentially expressed miRNAs in AD, using datasets deposited in the Gene Expression Omnibus (GEO). These findings were further validated through a systematic literature review. Pathway enrichment analysis was conducted to identify the key biological pathways that exhibited greater significance after random pathway analysis.

**Result:**

Through the LightGBM machine learning algorithm, we selected miRNAs from GEO data with the best predictive values and matched them with the miRNAs found in the systematic review. The systematic review identified 24 studies that met the inclusion criteria. The miRNAs common to both approaches were 30a‐3p, 193a‐5p, 143‐3p and 145‐5p, with reduced expression in AD patients compared to controls. The key biological pathways involving the genes regulated by these miRNAs included the TGF‐beta, ERBB signaling, MAPK pathways, and AP‐1 pathway.

**Conclusion:**

Our results suggest that miRNAs 30a‐3p, 193a‐5p, 143‐3p and 145‐5p are involved in biological pathways related to AD and represent potential biomarkers. TGF‐beta, ERBB and MAPK are pathways already related to AD in the literature. The novelty is the AP‐1 pathway, which regulates cell death and apoptotic factors, its activation decondenses genomic regions and activates cell death pathways in neurons. Furthermore, the increase in Aβ1‐42 elevates AP‐1 mRNA levels in astrocytes, inducing apoptosis. These findings underscore the importance of the miRNAs in AD and offer potential advancements in the understanding of its pathophysiology, mainly involving the AP‐1 pathway.

**Keywords**: Alzheimer's disease, miRNAs, cerebrospinal fluid.

**Acknowledgments**: CNPq e CAPES.

